# Neurobiological Basis of Aversion-Resistant Ethanol Seeking in *C. elegans*

**DOI:** 10.3390/metabo13010062

**Published:** 2022-12-31

**Authors:** Changhoon Jee, Enkhzul Batsaikhan, Chinnu Salim

**Affiliations:** Department of Pharmacology, Addiction Science and Toxicology (PHAST), College of Medicine, University of Tennessee Health Science Center, 71 S Manassas St., Memphis, TN 38103, USA

**Keywords:** ethanol preference, aversion-resistant seeking, *seb-3*, *fmo-2*, *C. elegans*

## Abstract

Persistent alcohol seeking despite the risk of aversive consequences is a crucial characteristic of alcohol use disorders (AUDs). Therefore, an improved understanding of the molecular basis of alcohol seeking despite aversive stimuli or punishment in animal models is an important strategy to understand the mechanism that underpins the pathology of AUDs. Aversion-resistant seeking (ARS) is characterized by disruption in control of alcohol use featured by an imbalance between the urge for alcohol and the mediation of aversive stimuli. We exploited *C. elegans*, a genetically tractable invertebrate, as a model to elucidate genetic components related to this behavior. We assessed the *seb-3* neuropeptide system and its transcriptional regulation to progress aversion-resistant ethanol seeking at the system level. Our functional genomic approach preferentially selected molecular components thought to be involved in cholesterol metabolism, and an orthogonal test defined functional roles in ARS through behavioral elucidation. Our findings suggest that *fmo-2* (flavin-containing monooxygenase-2) plays a role in the progression of aversion-resistant ethanol seeking in *C. elegans*.

## 1. Introduction

Alcohol use disorders (AUDs) are a significant public health concern. Alcohol accounts for 7.1% (for males) and 2.2% (for females) of the global burden of disease [1] and is also a leading cause of preventable death in the United States [2]. The adverse effects of alcohol abuse in humans have already been widely recognized. From the cellular toxicity of alcohol, which is toxic to most organ systems, and other risk factors of heart disease, stroke, liver disease, and digestive problems, to indirect contribution to injuries, such as via motor vehicle crashes and violence, various negative consequences and risks in many respects are well-known to many [1,3,4]. Nevertheless, the most noteworthy etiology of an AUD is characterized by loss of control over alcohol use despite recognition of these critical negative factors [5,6,7,8].

Aversion-resistant alcohol seeking behaviors have been studied in animals from nematodes to mammals as a model for this loss of control over alcohol use despite catastrophic consequences, modeling a medical condition of human AUD [9,10,11]. Aversion-resistant seeking (ARS) is characterized by an imbalance between the urge toward alcohol and disruption of control of alcohol use; however, the molecular mechanisms of how this loss of control progresses remain unclear. An ARS scale has been used in human genetic studies for reliable assessment of alcohol craving and dependence [12,13,14]. In a rodent model, aversion-resistant alcohol intake was investigated via pairing the intake with bitter quinine or a foot shock [9,10,15]. To better understand the underlying mechanisms of ethanol ARS, we exploited *C. elegans*, a genetically tractable invertebrate, as a model to elucidate genetic components related to ARS behavior. *C. elegans* has represented comparable physiological effects to humans at similar blood alcohol concentrations: acute functional tolerance, withdrawal symptoms, and induction of preference [16,17,18,19,20,21]. The ethanol preference of worms is elicited via prolonged exposure to ethanol. Furthermore, worms reproduce key features of mammalian alcohol-seeking behaviors: repeated attempts, endurance, and finally, ARS with an increase in ethanol preference [11]. 

ARS in *C. elegans* has been demonstrated through application of two distinct behavioral programs that conflict in the worm model: ethanol seeking and concurrent avoidance of aversive stimuli that block ethanol seeking [11]. The avoidance behavior of worms through the nociception process is directly related to the survival of the individual [22,23]. Thus, an ARS model of worms, using nociceptive stimuli, is ecologically suitable for modeling of persistent alcohol use despite aversive consequences in humans, which is increasingly recognized as a pivotal characteristic of an AUD [9,24,25,26]. 

In rodent models, aversion-resistant alcohol intake emerged after longer-term drinking [9,10,27,28,29]. In the worm model as well, prolonged exposure to ethanol resulted in transcriptome changes associated with physiological changes that are relevant to AUDs [30]. Previously, we assessed the transcriptional regulation of the neuropeptides involved in the progression of ARS in *C. elegans* at the system level. We have demonstrated that neuropeptides of the brain stress system interact in order to progress ARS [11]. The conserved role of the membrane lipids involved in development of AUDs [20], along with the specific role of these neuropeptides in stress responses including energy metabolism, highlighted the altered expression of genes involved in cholesterol metabolism in our ethanol-dependent worm model, and here, we propose the impact of cholesterol-metabolism coping with stress as a possible mechanism that affects susceptibility to neuroplasticity that is associated with ethanol preference and ARS. In this paper, we evaluate the role of cholesterol in progression of ARS. We have shown that the lipid environment modulates the induction of ethanol dependence in wild-type worms via showing alteration of the development of ARS preference and progression. We further evaluated this lipid involvement in worm ethanol dependence using susceptible dependent variants and additional variants that are involved in lipid metabolism. We preferentially selected molecular components thought to be involved in cholesterol metabolism, which is transcriptionally regulated by stress signals that mediate progression of ARS. Among these genes, which are differentially expressed in mutant strains that are susceptible to ethanol dependence, we verified the functional role of *fmo-2* (flavin-containing monooxygenase) in ARS; its mammalian orthologue is well-known as a central regulator of cholesterol balance. Our data support that cholesterol and the related pathways that regulate its homeostasis play a role in progression of ARS through neuropeptide signaling, and that the neurobiological basis of aversion-resistant ethanol seeking has been functionally conserved.

## 2. Results

*C. elegans* replicates alcohol-dependent-animal behavioral traits known from mammalian studies (such as enhanced acute functional tolerance, withdrawal symptoms, and preference). More explicitly, ethanol preference is elicited in wild-type (WT) *C. elegans* specimens that have experienced chronic ethanol exposure [11,19]. During chronic exposure to ethanol, multiple factors and physiological pathways are involved in ethanol-preference development and maintenance, which are important mechanisms of AUD progression. 

Cholesterol, like other lipids, is an essential component that is involved in many physiological processes, such as cell-barrier formation and signal transduction. Particularly, the nervous system has a rich lipid composition, so the human brain has both a high lipid content and high diversity [31,32]. It has been consistently suggested that microdomains in membranes known as lipid rafts may have a functional role in development of substance use disorders (SUDs). Additionally, in worms, it has been demonstrated that membrane lipids play an important role in development of acute functional tolerance of ethanol [20]. *C. elegans* is a cholesterol auxotroph. Although cholesterol is not synthesized, it is an essential component involved in many physiological processes in *C. elegans*, such as molting, reproduction, dauer formation, and metabolism [33,34,35]. These worms require a dietary supply of this sterol and contain a wide range of saturated, monounsaturated, and polyunsaturated fatty acids (PUFAs) similar to that in mammals [36,37,38]. Since worms obtain cholesterol from their diet (bacteria and the culture media in the laboratory), an environment in which sterol lipids are removed from biological membranes can be effectively established at the organism level. Hence, the characteristic of cholesterol auxotrophy was used to assess the role of membrane lipids in progression of ethanol dependence. 

**Figure 1:**
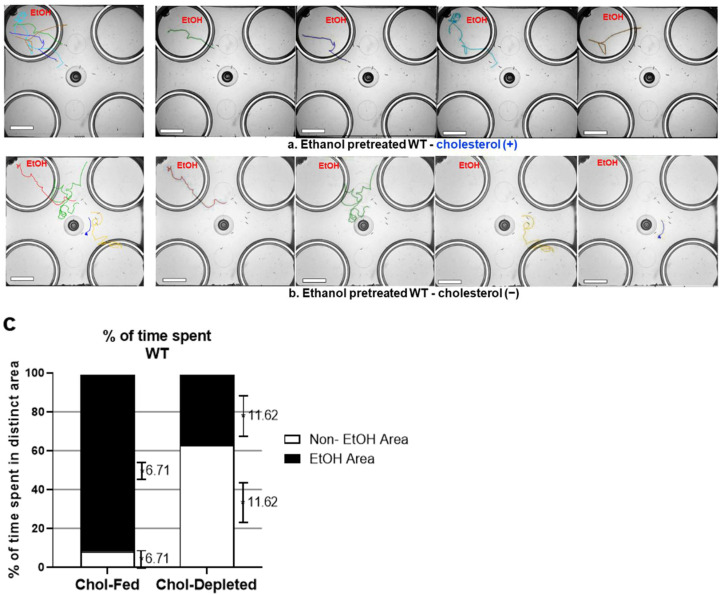
Cholesterol-depleted WT animals explored the nonethanol area even after ethanol pretreatment while cholesterol-fed animals headed straight to and remained in the ethanol area. (**a**) Trajectories of individual WT animals, cholesterol-fed (ethanol-pretreated for 4 h on 300 mM of ethanol). (**b**) Trajectories of individual WT animals, cholesterol-depleted (ethanol-pretreated for 4 h on 300 mM of ethanol). Ethanol-pretreated animals were placed in the middle of the assay plate, which contained ethanol (300 mM) only in the top left well (red EtOH). All wells were marginally covered by media that allowed free motion between the areas. Scale bar = 10 mm. (**c**) Behavioral quantification of (**a**,**b**). Ethanol-pretreated WT animals (cholesterol-fed) spent more time in the ethanol area, whereas cholesterol-depleted WT animals explored the nonethanol area more, even when ethanol-pretreated under the same conditions. These data were analyzed employing a chi-square test, which indicated df 66.42 and 1 z 8.150, *p* < 0.0001. Error bars shown to the right of each section (ethanol area or non-ethanol area) of the bar graph is SEM (N = 6, cholesterol-fed; N = 14, cholesterol-depleted).

It has been reported that triacylglycerol (TAG) levels influence development of acute functional tolerance (AFT) of ethanol in worms and showed that cholesterol depletion impaired AFT development [20]. Hence, we asked if cholesterol depletion altered development of ethanol preference as well. Cholesterol-depleted animals were tested in a free-moving preference assay on a four-well plate; only one of those wells contained ethanol (300 mM), as previously described [11]. The locomotion trajectories of each individual animal, exhibiting exploratory pattern, showed a distinct difference in the responses to ethanol between cholesterol-fed and cholesterol-depleted animals. In contrast to the WT animals that were fed cholesterol, the cholesterol-depleted animals showed no orientation toward the ethanol area after chronic exposure to ethanol and spent more time exploring the area without ethanol (Figure 1). Like the WT animals that were fed cholesterol, whose locomotory behavior was not damaged through the ethanol pretreatment, cholesterol-depleted animals in both naïve and ethanol pretreated groups had unimpaired locomotor behavior.

**Figure 2:**
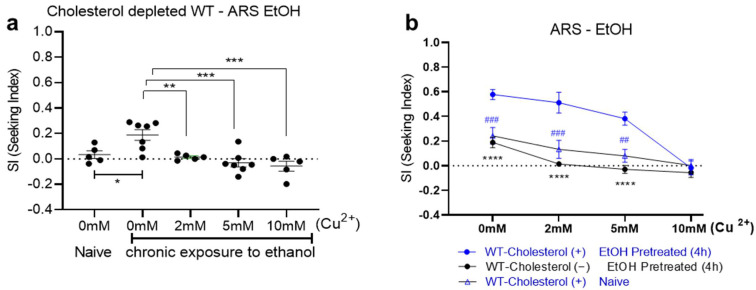
Behavioral quantification of ethanol preference and ARS of cholesterol-depleted WT animals (ARS: aversion-resistant seeking). (**a**) Strength of ethanol seeking is represented by the SI under different concentrations of a copper barrier (no barrier, 2 mM, 5 mM, and 10 mM). Compared to naïve WT animals grown in cholesterol-depleted conditions, cholesterol-depleted WT animals after ethanol pretreatment for 4 h developed ethanol preference. They showed mild chemotaxis to ethanol in the assay plate without an aversive barrier. However, few cholesterol-depleted WT animals crossed over aversive barriers. One-way ANOVA, *p* = 0.0002, F (4, 24) = 8.454, post hoc multiple comparison test; Dunnett’s (*p* < 0.05, *; *p* < 0.01, **; *p* < 0.001, ***). Each dot represents an assay that used a population of 100–150. (**b**) Data sets (**a**) from cholesterol-depleted animals were compared to those of the control group: cholesterol-fed WT animals. Cholesterol-fed WT animals developed ethanol preference and ARS after chronic exposure to ethanol, whereas the developments of ethanol preference and ARS were impaired in cholesterol-depleted WT animals. [Fcholesterol (1, 48) = 78.93, *p* < 0.0001; Fconc (3, 48) = 24.32, *p* < 0.0001; Fcholesterol x conc (3, 48) = 7.122, *p* = 0.0005]. N numbers were [0 mM = 10, 2 mM = 6, 5 mM = 6, 10 mM = 10 for WT cholesterol-fed; 0 mM = 7, 2 mM = 5, 5 mM = 7, 10 mM = 5 for WT cholesterol-depleted; and 0 mM = 6, 2 mM = 5, 5 mM = 6, 10 mM = 6 for WT cholesterol-fed naïve]. A two-way ANOVA comparison showed significant differences based on cholesterol feeding, barrier concentration, and the interaction of the two. Significant post hoc differences (multiple comparison correction using the Bonferroni method) between the cholesterol feedings (fed vs. depleted) at no barrier, the 2 mM barrier, and the 5 mM barrier are shown (*p* < 0.0001, ****). Significant post hoc differences (Bonferroni’s multiple comparison test) between naïve vs. ethanol-treated at no barrier, the 2 mM barrier, and the 5 mM barrier are shown (*p* < 0.01, ##; *p* < 0.001, ###).

We further investigated the behavioral changes caused via cholesterol depletion in the established measurement for the motivational strength of ethanol preference. In the ARS assay, we calculated the animals that had successfully reached ethanol over the aversive Cu^2+^ barrier in time, and the seeking index (SI) was obtained as described before [11]. The increased SI at the 0 mM (no barrier) represented sole ethanol preference while the increases at the 2 mM, 5 mM, and 10 mM barriers exhibited ARS. Reduced ethanol preference and ARS were observed in cholesterol-depleted WT animals. Cholesterol-depleted animals did not show preference for ethanol as much as did animals fed cholesterol after the ethanol experience, but also showed no complete defect in ethanol preference, as ethanol preference still appeared to develop. However, impaired ARS, which failed to cross the Cu^2+^ barrier for ethanol at low (2 mM, 5 mM) and high (10 mM) concentrations were identified (Figure 2). This is consistent with the observation in Figure 1 and similar to the SI values seen in the cholesterol-fed WT animals that were naïve before ethanol treatment (Figure 2b). 

**Figure 3:**
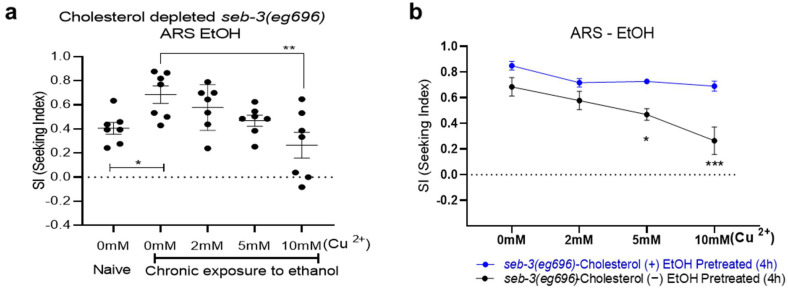
Cholesterol depletion suppressed the development of ARS in *seb-3 gf* animals that were susceptible to ethanol dependence (ARS: aversion-resistant seeking). (**a**) The cholesterol-depleted *seb-3(eg696)* animals developed ethanol preference after ethanol pretreatment for 4 h. One-way ANOVA, *p* = 0.0035, F (4, 30) = 4.948, post hoc multiple comparison test; Dunnett’s (*p* < 0.05, *; *p* < 0.01, **). Each dot represents an assay that used a population of 100–150. (**b**) Data sets (**a**) from cholesterol-depleted animals were compared to the control group: cholesterol-fed *seb-3(eg696)* animals. The cholesterol-depleted *seb-3(eg696)* animals demonstrated development of ethanol preference as much as did the animals that were fed cholesterol, whereas significantly reduced SIs in the 5 mM and 10 mM barriers represented ARS. [Fcholesterol (1, 40) = 26.29, *p* < 0.0001; Fconc (3, 40) = 6.210, *p* = 0.0015; Fcholesterol × conc (3, 40) = 1.811, *p* = 0.1607]. N numbers were [five in each cholesterol-fed *seb-3(eg696)* concentration and seven in each cholesterol-depleted *seb-3(eg696)* concentration]. A two-way ANOVA comparison showed significant differences based on cholesterol feeding, barrier concentration, and the interaction of the two. The Bonferroni method was used for multiple comparison correction as a post hoc test, and found significant differences in ARS (at 5 mM and 10 mM barriers) between cholesterol feedings (fed vs. depleted). *p* < 0.05, *; *p* < 0.001, ***.

Based on the results that showed that cholesterol depletion reduced development of ethanol preference and ARS in WT animals, we next asked whether cholesterol depletion affected ethanol preference even in mutant strains that were susceptible to ethanol dependence. In particular, we assessed a stress model in ethanol-dependent worms, in which the role of a mammalian orthologue was also involved in regulation of energy metabolism. *seb-3(eg696)* gain-of-function (*gf*) animals were shown to be susceptible to ethanol dependence, which was demonstrated by enhancement of all of the ethanol related behaviors of the worms: acute functional tolerance to ethanol, withdrawal-induced tremors, repeated attempts to seek ethanol, and finally, ARS [11,16]. Interestingly, in the *seb-3(eg696)* animals, cholesterol depletion prevented a greater progression of ethanol preference, which was significantly prominent in ARS. Cholesterol depletion suppressed the enhanced SI of the *seb-3(eg696)* animals in the ARS assay (Figure 3). The cholesterol-depleted seb-3*(eg696)* animals exhibited significantly low SI values compared to the cholesterol-fed *seb-3(eg696)* animals against higher-concentrated Cu^2+^ barriers (Figure 3). 

**Figure 4:**
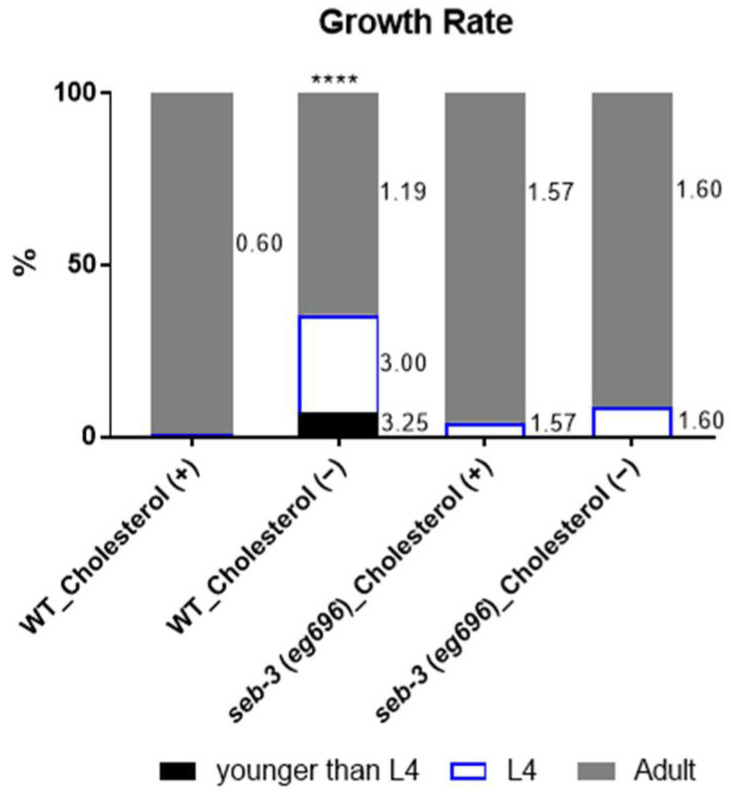
Cholesterol depletion altered developmental rates in WT *C. elegans*. The cholesterol depletion culture condition is demonstrated as the delayed growth rate that the WT animals should have shown. The number of animals in each developmental stage, after four days in embryos that were synchronized to the stage of birth for 1 h, is shown (younger than L4 stage, L4 stage, and adult). A total of 428 (WT-Chol_Fed), 628 (WT-Chol-depleted), 205 (*seb-3*-Chol_fed), and 189 (*seb-3*-Chol_depleted) worms from four replicates of biological samples were analyzed to obtain the average growth rate (%). The delayed growth-rate effect of cholesterol depletion was suppressed in the *seb-3(eg696)* variants. A chi-square test indicated **** *p* <0.0001. Error shown to the right of each section of the bar graph is SEM.

*C. elegans* requires sterol, supplied as cholesterol, in its diet. Cholesterol deprivation decreases brood size and delays growth to adulthood in the first generation of WT animals cultured under cholesterol-depleted conditions [39]. With 1-day adults obtained for each preference and ARS assay, the growth rate of the WT animals was confirmed for a control experiment under cholesterol depletion conditions, and only worms that reached adulthood (although delayed) were collected and used for the assay. Interestingly, we found that the *seb-3(eg696)* animals exhibited growth rates similar to those of the cholesterol-fed animals, overriding the delayed growth-rate effect of cholesterol depletion (Figure 4). Therefore, assuming that cholesterol depletion-induced ARS reduction in adult *seb-3*(*eg696*) worms is not due to a developmental defect, we postulated and pursued a possible pathway resulting from transcriptional regulation by the stress system during chronic ethanol exposure. 

Persistent upregulation of reward-seeking results in long-term dysregulation of neuronal gene expression profiles [40,41,42]. Recently, Bettinger et al. revealed that ethanol-induced transcriptional changes in worms underlie physiological responses that may contribute to AUDs [20,30]. They showed differentially expressed gene profiling in response to chronic exposure to ethanol. In our recent study, we analyzed the differentially expressed gene expression profiles of *seb-3*(*eg696*) animals, a genetic variant that exhibits exceedingly enhanced aversion-resistant ethanol seeking, and identified a total of 16 GO terms in upregulated and downregulated gene clusters [11]. Here, gene profiling from microarray datasets conducted previously [11] was reanalyzed and investigated as to whether improved algorithms could improve the level of agreement between more diverse platforms and identify candidates that were overlooked. We introduced an additional platform (g:Profiler: [43]) to reanalyze the data sets.

**Figure 5:**
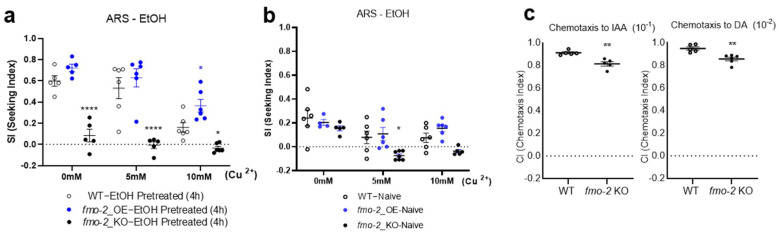
FMO-2 functions in modulation of ethanol preference and ARS. (**a**) *fmo-2*-overexpressing animals that were pretreated with ethanol for 4 h surmounted a stronger aversive barrier for ethanol. More *fmo-2*-overexpressing animals crossed over the barrier for ethanol (demonstrating ARS) than did the WT animals. The *fmo-2* KO animals showed impaired ethanol preference and ARS. Strength of ethanol seeking was represented by SIs under different concentrations of the copper barrier (no barrier, 5 mM, and 10 mM). A two-way ANOVA comparison of the strains over different barrier concentrations showed significant differences based on genotype, concentration, and the interaction of the two. [FGenotype (2, 41) = 65.25, *p* < 0.0001; FConcentration (2, 41) = 19.98, *p* < 0.0001; FGenotype × Concentration (4, 41) = 2.668, *p* = 0.0456]. Significant post hoc differences (multiple comparison correction using Dunnett’s method) between strains in each condition (no barrier, 5 mM, or 10 mM) are shown (*p* < 0.05, *; *p* < 0.0001, ****). Each dot represents an assay that used a population of 100–150. (**b**) Naïve animals were used as controls in the ARS assay. A two-way ANOVA comparison of the strains over different barrier concentrations showed no significant differences based on the interaction of genotype and concentration. [FGenotype × Concentration (4, 26) = 1.328, *p* = 0.2858]. Significant post hoc differences (multiple comparison correction using Dunnett’s method) were only observed between the *fmo-2* KO and the WT animals at a 5 mM barrier. *p* < 0.05, *. (**c**) Chemotaxis control of the *fmo-2* KO animals. Mann–Whitney (*p* < 0.01., **). IAA: isoamyl alcohol; DA: diacetyl.

Our unbiased transcriptional profiling linked the signal pathways involved in cholesterol homeostasis to alcohol-relevant behavior ARS. We identified altered expression levels of the genes involved in cholesterol balance in the *seb-3(eg696)* adults (Table 1). For further prioritization of candidate genes, human orthologues were identified using DAVID (The Database for Annotation, Visualization, and Integrated Discovery) [44,45], COBALT (a constraint-based alignment tool; https://blast.ncbi.nlm.nih.gov/Blast.cgi (accessed on 4 October 2022), Multiple Sequence Alignment version 1.19.1 and version 1.22.0) [46], and Ortholist2 [47]. In fact, *fmo-2*, the flavin-containing monooxygenase family, was the most upregulated candidate in quantitative analysis, as shown in Table 1. Mammalian FMOs are pleiotropic and have roles in NADPH-dependent oxidative metabolism of chemicals [48], including functions relevant to roles in regulation of energy homeostasis and metabolic aging [49,50]. In worms, activation of intestinal FMO-2 has been reported to promote longevity and health span downstream of HIF-1 (hypoxia-inducible factor 1) [51]. FMO-2 is predicted to be an orthologue of mammalian FMO5/FMO3 [52], which reorganizing cholesterol balance and signaling to lipid transporters such as ATP binding cassette (ABC) transporters [53]. Hence, along with the results shown in Figure 3 and Table 1, we assessed whether *fmo-2* variants are involved in ARS regulation. The animals with *fmo-2* overexpression, the functional role of which in longevity and health span was demonstrated by Leiser et al. [51], showed an enhanced SI at the 10 mM barrier, along with an upregulated graph pattern in overall concentration (Figure 5a, one-way ANOVA), consistently with the results shown in Figure 3 and Table 1, whereas knockout (KO) animals did not develop ethanol preference (0 mM barrier) after pretreatment as much as did the WT animals, or cross both low and high concentrations (5 mM, 10 mM) of the barrier in the ARS assay (Figure 5a). As a result of the control experiments that used naïve animals, aversive responses to Cu^2+^ stimuli were not defective in the *fmo-2*-overexpressing animals (Figure 5b), and thus, the *fmo-2*-overexpressing animals appeared to exhibit enhanced ethanol preference, which can override the interference of noxious stimuli after ethanol pre-exposure. To assess whether the defective development of ethanol preference in the *fmo-2* KO animals was ethanol-specific, chemotaxis was performed using isoamyl alcohol (IAA) and diacetyl (DA). Chemotaxis indexes were slightly decreased for both IAA and DA, however, the ability to detect IAA/DA and reach the IAA/DA area was intact. Accordingly, we concluded that *fmo-2* contributes to progression of ARS.

## 3. Discussion

ARS has been defined as repetitive attempts and achievement of ethanol seeking despite facing adverse barriers. It models the medical condition of a human AUD, characterized by an impaired ability to control alcohol use despite adverse consequences [54]. The progression of ARS in *C. elegans* is hypothesized to be imbalanced between enhanced ethanol seeking and loss of a controlling avoidance program. *C. elegans* has 32 presumed chemosensory neurons that detect a variety of olfactory and gustatory cues [23,55,56,57], and its avoidance programs are mediated by polymodal sensory neurons for nociception [23,58]. Ethanol exposure did not impair the ability to sense aversive stimuli in WT worms and reliably elicited preference and ARS. The *fmo-2*-overexpressing worms that were pre-exposed to ethanol represented a preference for ethanol in an environment in which ethanol was presented, and more animals readily crossed the aversive chemical barrier to reach the ethanol area. The *fmo-2*-overexpressing naïve animals in the ARS assay showed that their ability to detect aversive stimuli was still intact. Thus, enhanced ARS of *fmo-2*-overexpressing animals could be an endophenotype for compulsive-like ethanol seeking. Having defined enhanced ARS in overexpressing animals, we next investigated whether *fmo-2* functioned in modulation of ethanol preference and ARS. We tested whether the developmental impairment of ethanol preference in *fmo-2* KO animals was ethanol-specific or whether knockout of the fmo-2 gene generally disrupted chemotactic responses to volatile odorants. *C. elegans* is intrinsically attracted to certain odors in a concentration-dependent manner [59]. We evaluated the chemotaxis ability appropriately in *fmo-2* KO animals using volatile attractant odorants with chemotaxis mediated by distinct neurons and pathways. We analyzed the chemotaxis to the volatile attractant odorant isoamyl alcohol (IAA), which is detected and mediated by AWC olfactory sensory neurons, and to diacetyl (DA), which is mediated by AWA sensory neurons [55]. Although a slight reduction was observed in the chemotaxes to both IAA and DA, sensory perception and subsequent chemotaxes were intact. Consequently, our data suggest that *fmo-2* is required for ethanol preference and ARS elicited via prolonged exposure to ethanol.

*fmo-2* has been reported as an FMO in *C. elegans*, and activation of *fmo-2* has been demonstrated to promote longevity and health span in hypoxic responses or under dietary restriction [51]. It is known that there are five FMOs in both mammals and *C. elegans*, all of which have been derived from a single ancestral FMO [60]. FMO5/FMO3, the mammalian orthologue of FMO-2, are the major forms of FMOs in livers of mammals [61,62] and have been known to play important roles in regulation of cholesterol and fat metabolism [49,63], in particular reorganizing cholesterol balance and signaling to lipid transporters such as ATP-binding cassette (ABC) transporters [54]. ARS in *C. elegans* has been demonstrated to be facilitated and progressed by coordination and interactions between conserved neuropeptide signaling, which is also involved in regulation of energy metabolism and fat deposition [64,65]. Cholesterol, known as a structural component of cell membranes, has recently emerged as a direct major regulator of ion channel function. Cholesterol has also been shown to indirectly regulate channel function in a way that affects the biophysical properties of the membrane. In our expression profiling analysis, using our stress-induced ethanol-susceptible model, molecular components with significant associations in both direct and indirect regulation via cholesterol were identified. We therefore hypothesized that cholesterol metabolism in response to stress signals may contribute to the progression of ARS indirectly via regulation of the microdomain of the membrane and directly via specific ion channels. Assuming roles in the nervous system, it may occur in state-dependent integration of multisensory information during mediation of ethanol perception and mediation of aversive stimuli. Accordingly, this further highlights the need to investigate whether cholesterol metabolic pathways play an important role in progression of ARS. However, since *fmo-2* is expressed in diverse tissues, such as intestinal tissues, as well as in neuronal cells, specific tissues that rescue defective ethanol preference and ARS in *fmo-2* KO animals or specific tissues in which overexpression enhances ethanol preference and ARS will be important for further investigation. 

Recently, transcriptional regulation of genes involved in cholesterol homeostasis during persistent neural adaptation of alcohol consumption in rodent models has been reported in specific brain regions [66]. Chronic alcohol consumption with forced abstinence was reported to lead to altered expressions of genes involved in cholesterol synthesis, transport, and degradation. Furthermore, among these genes, ABCA1 was downregulated via CRF signaling in the initial critical stage of atherosclerosis [67]. Accumulation of cholesterol in arterial macrophages is pivotal during the pathogenesis of atherosclerotic cardiovascular disease. ABC transporter families, such as ABCA1 and ABCG1, play important roles in this modulation [68], and ABCA1 is extensively studied in relation to the reverse cholesterol pathway and cellular cholesterol homeostasis, while other ABC transporters have been studied in relation to multidrug resistance in tumor cells [69]. An increase in active ABCA1 in the plasma membranes of Baby hamster kidney (BHK) cells causes a redistribution of cholesterol, sphingomyelin, and caveolin, resulting in an expanded nonraft membrane fraction [70]. 

Cholesterol metabolism has been reported to be perturbed by various types of stress. For examples, cholesterol metabolism is perturbed by amyloid-beta-induced, membrane-associated oxidative stress in the pathogenesis of Alzheimer’s disease (AD) [71]. ER stress contributes to dysregulation of lipid metabolism [72]. Interestingly, psychological stress, such as chronic social defeat stress, also induces unfavorable lipid profiles when combined with a high-fat diet [73]. Importantly, the molecular components of the pathways involved in this cholesterol homeostasis and the neuropeptide signaling that regulates it are well-conserved from nematodes to humans. 

Our findings support the mechanism of regulation of cholesterol homeostasis via neuropeptide signaling, as described above. We have shown that lipid environments modulate induction of ethanol dependence in worms. Our data suggest conservation of the functional role of *fmo-2* (flavin-containing monooxygenase) in progression of ARS, suggesting a possible mechanism of ARS along with a role of ionic factors in reorganization and reinforcement of lipid-membrane microdomains via downregulation of ABC transporter orthologues, as shown in Table 1. In particular, it is noteworthy here that the decrease in ARS by cholesterol depletion in *seb-3*(*eg696*) animals did not completely impair ARS, but was restored to the extent that developed in WT animals. Two distinct but closely related regulatory processes of cholesterol metabolism in response to ethanol may be suggested, which directly or indirectly affect the biophysical properties of the membrane, either regulation of the gating property of the channels or regulation of the microdomains of the membranes. Considering the pathological aspect of AUD patients, which uncontrolled alcohol use despite negative consequences, further investigation of the effect of ethanol on lipid metabolism will provide more diverse and effective intervention for the treatment of AUDs.

## 4. Materials and Methods

All strains were maintained on nematode growth media (NGM) plates with *Escherichia coli* (OP50) at 20 °C [74], and the hermaphrodite thereof was used for behavioral analysis. The wild-type animals used for the experiment were of the Bristol N2 strain. *seb-3*(*eg696*) *gf* had been previously isolated from our genetic screening [16]. The VC1668, *fmo-2 (ok2147)* and KAE10, seaSi40 [(pCFJ448) (*eft-3p::fmo-2* + H2B::GFP) + *Cbr-unc-119*( + )] I strains, which validated as *fmo-2* KO and *fmo-2*-overexpressed in [51], were obtained from Caenorhabditis Genetics Center (CGC, Minneapolis, MN, USA).

### 4.1. Cholesterol-Depleted Animals

WT or seb-3(eg696) animals were cultured in completely cholesterol-depleted NGM provided with Escherichia coli (OP50) grown directly in sterol-free media and were termed cholesterol-depleted animals. Complete cholesterol deprivation was achieved following the protocol demonstrated before, in [39]. Bacterial and *C. elegans* media had to contain 3.5 mM of Tris.Cl, 2 mM of Tris, 34 mM of NaCl, and, importantly, 3.1 g/L of ether-extracted peptone. The ether-extracted peptone was prepared in a large beaker in a fume hood. Peptone powder was mixed with an excess volume of ether, allowed to settle, and decanted, and the process was repeated twice more. The extracted peptone was allowed to dry overnight in the hood to remove the remaining ether. For the growth rate of cholesterol-depleted animals, WT 1-day gravid adult animals were allowed to lay eggs for 1 h either on the NGM that contained cholesterol or the cholesterol-depleted NGM seeded with cholesterol-depleted OP50, and then the adults were removed to produce synchronized embryos. After 4 days at 20 °C, the stages of the worms were defined and counted based on characteristics at each stage, as described in [39,75,76]. Worms determined to be in the gravid adult stage were used for further assays of ethanol preference and ARS.

### 4.2. Trajectory Analysis of C. elegans Locomotion in the Free-Moving Ethanol Preference Assay on a 4-Well Plate

This was conducted as described before, in [11]. All 4 wells and the other regions (1.9 cm^2^) were filled up equally, to the top, with NGM. Ethanol was added to only one of the four wells to a concentration of 300 mM. One-day adult animals were trained to develop ethanol preference, as described in [11,19]. Thirty-minute locomotion was recorded, and trajectories were analyzed with Wormlab software Ver. 2020 (MBF Bioscience, Williston, VT, USA). 

### 4.3. Aversion-Resistant Seeking (ARS) of Ethanol Assay

Ethanol pretreatment was conducted as described in [16,17,18]. Simply summarized, OP50 was seeded to a half-region of an NGM plate 3 days prior to the ethanol treatment. For cholesterol-depleted animals on ethanol, cholesterol-depleted OP50 was seeded in the same way on the cholesterol-depleted plate. Ethanol was added to the unseeded region and allowed to diffuse for 2 h; the plates were sealed with parafilm to keep the ethanol from evaporating. One-day adult animals were incubated on a 300 mM ethanol plate with OP50 for 4 h and then trained worms were used for ethanol preference and ARS assay, which was conducted as described before in [11]. A chemotaxis assay was conducted, as described. Forty minutes later, the number of animals in each marked section (A, ethanol region; B, opposite of ethanol region; C, crossing barrier toward ethanol region; and D, not crossing barrier toward B region) was counted to calculate the seeking index. This index was calculated with [(number of animals in A−number of animals in B)/total number of animals [seeking index SI = (A−B)/Total (A+B+C+D)]. The SI in each trial was obtained from an assay of a population of 100 to 150.

### 4.4. Statistical Analysis

The mean and the standard error of the mean (SEM) were determined for each experimental parameter. The data were analyzed employing a chi-square test or an ANOVA (GraphPad Prism version 8.0.1). Values below 0.05 were considered to be significant.

### 4.5. Gene Ontology Enrichment Analysis

Previously, microarray analysis had revealed 716 transcripts that are differentially expressed in *seb-3*(*eg696*) *gf* animals (≥ 1.5×) compared to WT animals, as described before. Gene ontology enrichment analysis was conducted in an additional platform (g:Profiler: [44]), which had been updated with new data from Ensemble and Wormbase ParaSite, to reanalyze the data sets. Additionally, we identified the human orthologues that corresponded to prioritized, differentially expressed genes, using the gene ID conversion tool in the DAVID (Database for Annotation, Visualization, and Integrated Discovery) [44,45], the g:Profiler gene ID conversion tool [43], and Ortholist2 [47]. Subsequently, gene ontology enrichment analysis was conducted in DAVID and g:Profiler, using the data set of orthologous candidates. Based on the ethanol preference shown by cholesterol-depleted worms and the significance of the lipid environment of the membranes in ARS, among the clusters identified via DAVID and g:Profiler analysis, the lipid-metabolism-process cluster (GO:0006629) was highlighted, and associated genes are shown in Table 1. Furthermore, we also analyzed data from FUdR-untreated samples [WT vs. *seb-3(eg696)*], used only to confirm that FUdR treatment in previous studies did not affect the expression profiling of WT animals (e.g., WT vs. WT-FUdR); additional candidate genes (marked as *) for lipid transporters are shown in Table 1. Previous studies analyzed pharmacologically germline-inhibited samples [5′-fluorodeoxyuridine (FUdR) treatment] to improve the chances of identifying differentially expressed genes in somatic cells, including neuronal tissue. The candidate genes in Table 1 were further analyzed using COBALT (a constraint-based alignment tool) [46]. 

### 4.6. Chemotaxis Analysis

Chemotaxis assays were performed as described in [55]. Briefly, chemotaxis plates were prepared with 10cm Petri dishes that contained 10 mL of assay agar (2% agar, 5 mM of KPO_4_ [pH6], 1 mM of CaCl_2_, 1 mM of MgSO_4_). The worms were washed twice in S-basal and once in distilled water and placed onto spots on the plates. A 1 µL amount of 1:10 IAA:EtOH was placed on one end test spot of each chemotaxis assay plate (10 cm), and 1 µL of ethanol was applied to the opposite endpoint as control. An amount of 1 µL of 1 M NaN3 was applied to immobilize the worms when they reached each spot. After one hour of chemotaxis, the animals were counted. The assay of chemotaxis to DA (1 µL of 1:100 DA:EtOH) was performed in the same way.

## Figures and Tables

**Table 1:** ABC transporters and *fmo-2*, which are differentially expressed genes in *seb-3(eg696) gf* animals, are prioritized to be involved in cholesterol homeostasis.

Altered	Gene ID	Gene	FoldChange	FDR	*p*_Value	Description	Human Orthologue
Up	WBGene00001477	*fmo-2*	20.97	0.05	0.04	Flavin containing monooxygenase	FMO5 (ENSG00000131781), FMO3 (ENSG00000007933)
Down	WBGene00003995	*pgp-1*	5.04	0.04	0.04	ABC transporter family (ABCB)	ABCB1 (ENSG00000085563), ABCB11 (ENSG00000073734), ABCB4 (ENSG00000005471)
Down	WBGene00004002	*pgp-8*	2.65	0.05	0.04		ABCB1(ENSG00000085563), ABCB11 (ENSG00000073734), ABCB5 (ENSG00000004846)
Down	WBGene00004003	*pgp-9*	2.42	0.01	0.00		ABCB1(ENSG00000085563), ABCB11 (ENSG00000073734), ABCB4 (ENSG00000005471)
Down	WBGene00001817	*haf-7*	2.05	0.01	0.00		ABCB9 (ENSG00000150967), TAP1 (ENSG00000168394)
Down *	WBGene00004004	*pgp-10*	2.34	0.05	0.02		ABCB1 (ENSG00000085563), ABCB4 (ENSG00000005471)
Down *	WBGene00004005	*pgp-11*	2.26	0.05	0.02		ABCB1 (ENSG00000085563), ABCB11 (ENSG00000073734), ABCB5 (ENSG00000004846)
Down *	WBGene00015479	*wht-1*	1.60	0.05	0.03	ABC transporter family (ABCG)	ABCG1 (ENSG00000160179), ABCG4 (ENSG00000172350)
Down *	WBGene00000023	*abt-5*	2.53	0.05	0.01	ABC transporter family (ABCA)	ABCA1 (ENSG00000085563), ABCA3 (ENSG00000167972), ABCA12 (ENSG00000144452), ABCA1 (ENSG00000179869)

* indicates the differentially expressed gene candidates indentified without FUDR treatment.

## Data Availability

All genetic variants used in this study can be obtained from Caenorhabditis Genetics Center (CGC, Minneapolis, MN, USA).
